# Competence and Readiness of Physicians in Saudi Arabia in Managing In-Flight Medical Emergencies: A Systematic Review

**DOI:** 10.7759/cureus.117148

**Published:** 2026-09-29

**Authors:** Muhannad Almutairi, Tariq Alharbi, Mohammed A Aljunaid, Sadeem Alharbi, Shahad E Alsulami, Nawwaf Albishi, Ziyad Alhatimi, Saud Khared, Abdulwahab Alharbi

**Affiliations:** 1 Family Medicine, Jeddah First Health Cluster, Jeddah, SAU; 2 Medicine, College of Medicine, University of Jeddah, Jeddah, SAU; 3 Family and Community Medicine, University of Jeddah, Jeddah, SAU; 4 Family Medicine, College of Medicine, University of Jeddah, Jeddah, SAU; 5 Medical Intern, College of Medicine, University of Jeddah, Jeddah, SAU; 6 Family Medicine, College of Medicine, University of Jeddah, Jedda, SAU

**Keywords:** aviation medicine, in-flight medical emergencies, medical education, physicians, readiness, saudi arabia, systematic review

## Abstract

As air travel in Saudi Arabia continues to grow rapidly, ensuring that physicians are competent and ready to manage in-flight medical emergencies (IMEs) is important. Evidence regarding physicians' competence and preparedness remains limited. This review aimed to assess physicians' competence, including knowledge, awareness, attitudes, confidence, and willingness, and readiness, including awareness of aviation physiology and onboard medical resources, and to identify key gaps and predictors of performance. A systematic search of PubMed, Scopus, Web of Science, and Google Scholar was conducted for studies published up to May 15, 2026. Cross-sectional studies involving licensed physicians in Saudi Arabia were included. Outcome measures included knowledge, awareness, attitudes, confidence, willingness, preparedness, and practices regarding IMEs. Risk of bias was assessed using the Joanna Briggs Institute (JBI) critical appraisal tool. This systematic review was conducted in accordance with the 2020 Preferred Reporting Items for Systematic Reviews and Meta-Analyses (PRISMA) guidelines and followed a predefined protocol registered in the International Prospective Register of Systematic Reviews (PROSPERO) (CRD420261328551), to ensure methodological rigor and transparency. Six cross-sectional studies from different regions of Saudi Arabia were included. Adequate knowledge of aviation physiology, onboard equipment, and emergency protocols ranged from approximately half to three-quarters of participants, and considerable variability was observed across studies. Most physicians expressed positive attitudes toward volunteering during IMEs, yet many lacked confidence due to insufficient targeted aviation medicine training, medicolegal liability concerns, and unfamiliarity with airline procedures. Physicians who had previously encountered an IME were more likely to have completed life-support training, to travel frequently, and to demonstrate higher competence and readiness to assist. Systemic barriers, including ambiguous legal frameworks and lack of formal aviation medicine training, further hindered effective emergency response. Despite a high willingness to assist during IMEs, doctors in Saudi Arabia have significant knowledge, confidence, and preparedness gaps. Establishing structured simulation-based training, enhancing legal structures, and furthering professional collaboration may help bridge these gaps. It is also recommended to develop a nationally unified framework and incorporate emergency training in medical education.

## Introduction and background

In 2023, global air travel volume reached 4.27 billion passengers, according to data from the World Bank [1]. While air travel is statistically safe, the immense scale of air travel brings into focus in-flight medical emergencies (IMEs), which refer to sudden medical events that happen during commercial flights and require either the use of emergency equipment on board or medical intervention [2]. When the established global incidence rate of 18.2 events per million passengers is applied to current travel volumes, over 77,000 medical emergencies occur at altitude annually [1,3]. Operationally, estimates suggest a global incidence of approximately one IME for every 604 commercial flights [4]. This emphasizes a critical need for traveling healthcare providers to be competent and ready to manage life-threatening conditions within the medically austere environment of a pressurized cabin. The primary catalyst for these emergencies is the unique physiological stressors encountered during air travel [3]. Commercial aircraft cabins are typically pressurized to an atmospheric equivalent of 8,000 feet (2,438 meters), where the barometric pressure is significantly lower than at sea level. This pressure differential causes arterial oxygen pressure to decrease from a baseline of 95-98 mmHg to approximately 55-56 mmHg and a drop in oxygen saturation to roughly 90% [4,5], creating a hypoxic environment that can exacerbate preexisting conditions or trigger acute symptoms even in otherwise healthy passengers [4].

A review of 14 international studies, encompassing 49,100 reported cases, showed that syncope was the most prevalent emergency (32.7% of cases), followed by gastrointestinal complaints (14.8%) and respiratory distress (10.1%). Although cardiovascular symptoms represent approximately 7.0% of total incidents [6], these cases are challenging because they can indicate life-threatening conditions such as acute coronary syndrome, pulmonary embolism, or pneumothorax.

These incidents carry significant operational weight [3]. While only 7.3% of emergencies result in diversion, this rate increases to 58% for cardiac arrests [4], with costs exceeding $893,000 per diversion and a rate of 11.1 per 100,000 flights [7]. It is crucial that physicians promptly evaluate and manage patients in these medically austere environments. This capability is essential for passenger safety and airline efficiency [4,6].

When an IME occurs, historical data suggest that up to 70% of all in-flight incidents are managed by cabin crew alone [8], while more recent evidence indicates that when a call for assistance is made, traveling physicians voluntarily step forward in 48% of cases [9]. Despite this high rate of volunteerism, the aircraft cabin remains a uniquely austere clinical environment characterized by limited space, persistent loud noise, and physiological changes resulting from lower barometric pressure [10]. Such conditions make it challenging to perform standard clinical procedures; for instance, loud noise can prevent clinicians from using a stethoscope to auscultate blood pressure manually [11]. There are no universal international training standards for IMEs [6]. Legal obligations also differ widely; the United States, England and Canada do not require physicians to assist, while Australia and several European countries impose a legal mandate to help [3].

Saudi Arabia's expanding aviation industry has raised the need to assess how prepared local physicians are. Several cross-sectional studies have attempted to evaluate willingness, confidence, and knowledge of various healthcare cohorts in the Kingdom, including primary care physicians [12,13]. These studies highlight the importance of IME training within the Saudi healthcare system. Despite these efforts, the existing evidence remains fragmented across different regions and specialties. To date, no systematic review has comprehensively synthesized this evidence to provide a unified assessment of physicians' competence and readiness in Saudi Arabia.

This review aimed to assess physicians' competence, including knowledge, awareness, attitudes, confidence, and willingness in managing IMEs, and readiness, including awareness of aviation physiology and onboard medical resources, and to identify key gaps and predictors of performance. By synthesizing available evidence, the review seeks to identify critical training gaps and sociodemographic or occupational factors that predict clinical preparedness. The findings from this review are designed to help shape standardized medical curricula and establish national training protocols. These measures aim to improve both the safety and quality of medical care delivered in the Saudi aviation sector.

## Review

Methods

Registration

This systematic review was conducted in accordance with the 2020 Preferred Reporting Items for Systematic Reviews and Meta-Analyses (PRISMA) guidelines [14]. The review followed a predefined protocol registered in the International Prospective Register of Systematic Reviews (PROSPERO) under registration number CRD420261328551, to ensure methodological rigor and transparency.

Study Eligibility Criteria

Eligibility criteria were defined based on the study objectives. Studies were included if they involved licensed physicians or medical residents practicing in Saudi Arabia; were cross-sectional or survey-based observational studies; assessed competence or readiness in managing IMEs, including knowledge, awareness, attitudes, confidence, willingness and preparedness; were published in English; and reported methodology and relevant outcomes clearly. Studies were excluded if they involved only medical students; were conducted outside Saudi Arabia; were case reports, reviews, editorials, letters, or conference abstracts; did not assess IME-related competence or readiness; were non-English publications; or lacked sufficient methodological detail.

Literature Search Strategy

A comprehensive literature search was conducted using PubMed, Google Scholar, Scopus and Web of Science to identify relevant studies published up to May 15, 2026. The PubMed search strategy used a combination of Medical Subject Headings (MeSH) and keywords with Boolean operators, and the strategies used in each database were as follows.

PubMed: ("in-flight medical emergencies" OR "inflight medical emergencies" OR "air travel" OR "aviation medicine") AND (knowledge OR attitude OR confidence OR preparedness OR willingness OR practice* OR competence OR readiness) AND (physician* OR doctor* OR resident* OR "medical resident") AND ("Saudi Arabia" OR KSA).

Google Scholar: "in-flight medical emergencies" "Saudi Arabia" physicians competence readiness.

Scopus: TITLE-ABS-KEY ("in-flight medical emergenc*" OR "inflight medical emergenc*" OR "in-flight emergenc*" OR "aviation emergenc*" OR "aeromedical emergenc*") AND (competence OR readiness OR preparedness OR knowledge OR attitude* OR confidence OR willingness OR practice*) AND (physician* OR doctor* OR "medical doctor*" OR resident* OR "general practitioner*") AND ("Saudi Arabia" OR "KSA" OR "Kingdom of Saudi Arabia").

Web of Science: TS = ("in-flight medical emergenc*" OR "inflight medical emergenc*" OR "in-flight emergenc*" OR "aviation emergenc*" OR "aeromedical emergenc*") AND (competence OR readiness OR preparedness OR knowledge OR attitude* OR confidence OR willingness OR practice*) AND (physician* OR doctor* OR "medical doctor*" OR resident* OR "general practitioner*") AND ("Saudi Arabia" OR "KSA" OR "Kingdom of Saudi Arabia").

Manual screening of the reference lists of all included studies was performed to identify any further relevant articles. Grey literature, dissertations, and conference proceedings were not searched, and eligibility was restricted to English- language publications. The search strings were deliberately broad, combining aviation-emergency terms with generic competence terms and no restriction on study design; the small number of retrieved records therefore reflects a genuinely narrow national evidence base on this topic rather than a restrictive search strategy. No updated search was run after 15 May 2026.

Study Selection and Screening

All identified records were imported into Rayyan, where duplicate records were removed. A two-stage screening process was conducted, comprising title and abstract screening by two independent reviewers followed by full-text screening for eligibility. Reasons for exclusion at the full-text stage were documented. Any disagreements between reviewers were resolved through discussion; if consensus could not be reached, a third reviewer was consulted. Inter-rater agreement was not formally quantified; a chance-corrected statistic such as Cohen's kappa was not calculated, and reviewer disagreement was instead handled by discussion and third-reviewer adjudication.

Data Extraction

Data extraction was independently performed by two reviewers using a standardized, pre-designed extraction form. The following variables were extracted: author or authors, year of publication, and study location; study design; sample size and participant demographics; physician specialty, such as emergency medicine, family medicine, or intensive care unit (ICU); level of training, comparing residents with practicing physicians; domains assessed, comprising knowledge, attitudes, confidence, preparedness, willingness and practices; measurement tools and scoring systems; key findings; and factors associated with higher competence or readiness, such as prior training or experience with IMEs. Discrepancies were resolved through discussion or by consultation with a third reviewer.

Quality Assessment (Risk of Bias)

The methodological quality of included studies was independently assessed by two reviewers using the Joanna Briggs Institute (JBI) Critical Appraisal Checklist for Analytical Cross-Sectional Studies [15]. Disagreements were resolved through discussion or consultation with a third reviewer. Each of the eight JBI items was rated as “yes”, “no”, or “unclear”. Studies with at least six of eight “yes” ratings (≥75%) were classified as having a low risk of bias, four to five (50-74%) as moderate, and three or fewer (<50%) as high. No included study met the high-risk threshold. No studies were excluded based solely on their quality assessment score; the risk of bias was utilized to provide context and evaluate the strength of evidence during narrative synthesis.

Data Synthesis

A meta-analysis was initially considered; however, due to heterogeneity in study populations, assessment tools, and outcome reporting, quantitative pooling was not feasible. Therefore, narrative synthesis was conducted. Findings were systematically grouped and analyzed across the following domains: knowledge of IMEs and aviation medicine; attitudes and willingness to assist during IMEs; confidence in managing IMEs; preparedness, including awareness of onboard medical resources and protocols; and practice-related responses, including prior experience with IMEs. Findings were compared across physician specialties, training level, comparing residents with practicing physicians, and prior exposure or training, such as basic life support (BLS), advanced cardiovascular life support (ACLS), and IME-related education. Key patterns, consistencies, and discrepancies across studies were identified and synthesized narratively.

Results

Screening of the Included Studies

The comprehensive search yielded 36 records in PubMed, Scopus, Google Scholar and Web of Science databases. After excluding duplicates, 23 records were screened based on titles and abstracts, resulting in the exclusion of 17 articles. Subsequently, six full-text articles were assessed for eligibility, after which no articles were excluded based on pre-specified criteria. Finally, six studies were included in the systematic review after inclusion criteria were met [12,13,16-19]. A detailed account of the identification, screening, eligibility, and inclusion process is presented in the PRISMA 2020 flow diagram (Figure 1).

**Figure 1:**
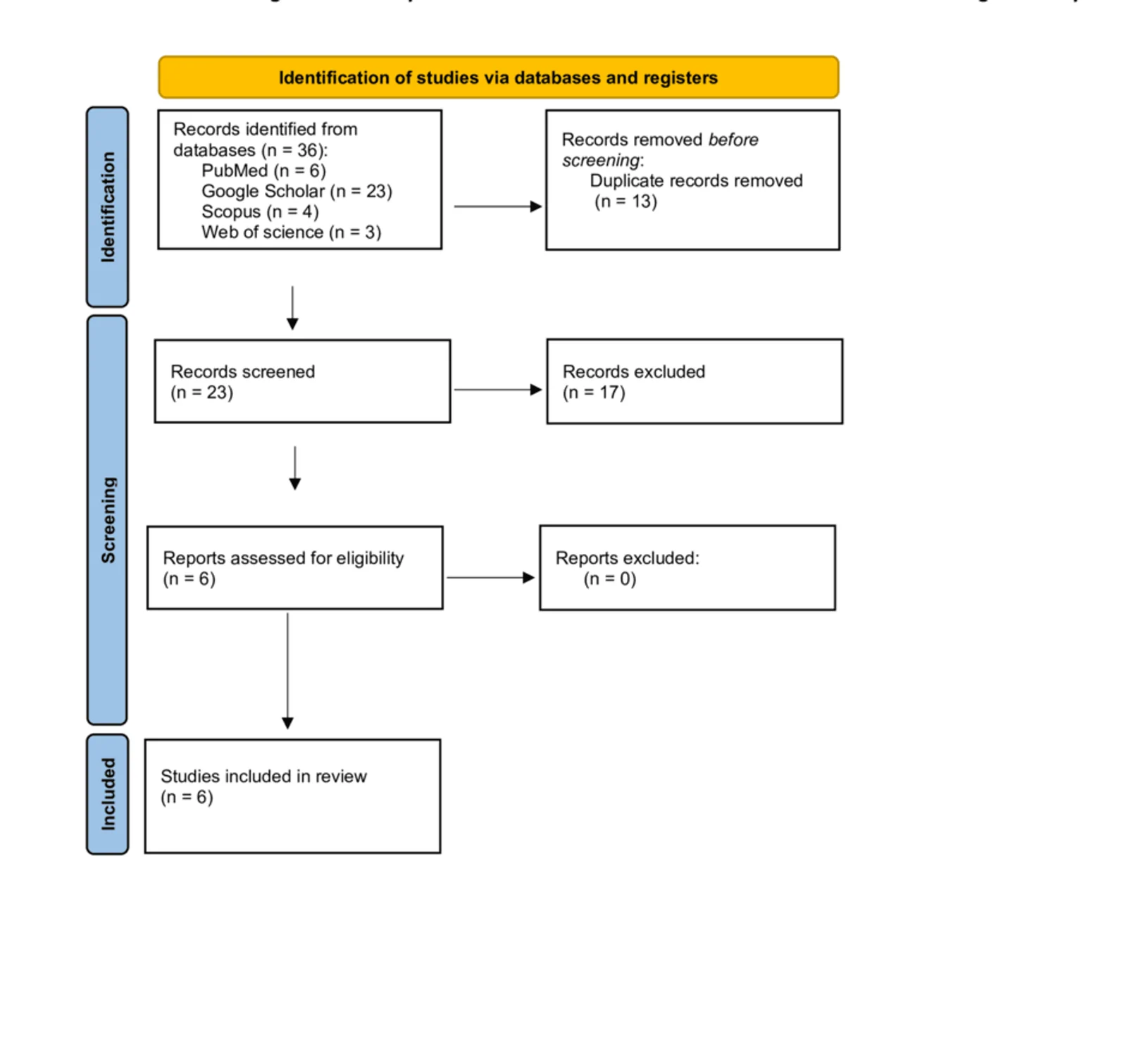
PRISMA chart of the included studies.

Characteristics of Included Studies

Six articles investigating the competence and readiness of physicians in Saudi Arabia in managing in-flight medical emergencies were included in our study. The included studies comprised cross-sectional studies. The origin of the studies was multiple regions, including Riyadh and Qassim. Sample sizes were obtained through diverse sampling methods such as stratified convenience sampling and simple random sampling. The sample sizes ranged from 155 to 5,000 participants. The mean ages of participants varied from 27 to 45 years. The summary characteristics of the included studies are reported in Table 1, and the baseline characteristics of the included studies are reported in Table 2. Given the wide variation in sample size (n = 155 to n = 5,000), in geographical coverage (single-region versus multi-region), and in target population (family medicine residents only versus all specialties), percentages are not directly comparable between studies and are presented descriptively rather than as equivalent estimates of the same underlying population.

**Table 1:** The summary characteristics of the included studies. SCFHS: Saudi Commission for Health Specialties; MOH: Ministry of health; SCHS: Saudi Commission for Health Specialties; PHCC: Primary Health Care Corporation; FM: Family medicine

Domain	AlShamlan et al. (2021) [18]	Alarifi & ALRowais (2023) [13]	Hussien et al. (2024) [19]	Almadhyan et al. (2025) [17]	Hawati et al. (2025) [16]	Almutairi & Sulaiman (2025) [12]
Country / Region	Saudi Arabia – multi-region	Saudi Arabia – Riyadh	Saudi Arabia – multi-region	Saudi Arabia – 5 regions	Saudi Arabia – multi-region	Saudi Arabia – Qassim region
Study Design	Cross-sectional online survey	Cross-sectional survey	Cross-sectional online survey	Cross-sectional study	Cross-sectional web-based survey	Descriptive cross-sectional study
Study Period	January 2021	January 2023	Not specified	March – September 2024	June – October 2024	2024
Data Collection Tool	Self-administered online questionnaire; 11 Likert items; adapted from Katzer et al. [20] & Ng et al. [21]	Self-administered questionnaire; adapted from Katzer et al. [20] & Ng et al. [21]	Structured online questionnaire; 14 items; adapted from AlShamlan et al. [18]; Cronbach α ≥ 0.70	Self-administered questionnaire; pre-validated (Ng et al. [21]); Google Forms	Comprehensive online survey (Google Forms); clinical scenarios + aviation physiology + Likert scale	Self-administered online questionnaire; developed from literature review; pilot tested on 20 participants
Sampling Method	Online link via SCFHS registered emails; no duplicate responses allowed	Online Google Form via SCFHS emails distributed to all FM residents across 8 programs	Stratified convenience sampling across regions and specialties via electronic medical communities	Randomised sampling from MOH & SCHS directories; online link (no duplicates)	Online survey distributed via email and face-to-face by data collectors	Simple random sampling of PHCCs; convenient non-probability sampling

**Table 2:** The baseline characteristics of the included studies. FM: Family Medicine; EM: Emergency Medicine; PHC: Primary healthcare; PGY: Postgraduate Year; GP: General practitioner

Domain	AlShamlan et al. (2021) [18]	Alarifi & ALRowais (2023) [13]	Hussien et al. (2024) [19]	Almadhyan et al. (2025) [17]	Hawati et al. (2025) [16]	Almutairi & Sulaiman (2025) [12]
Target Population	All physicians in KSA (all specialties)	Family medicine (FM) residents in Riyadh	Medical interns & postgraduate physicians in KSA	Licensed physicians across 5 regions of KSA	Emergency medicine, family medicine & ICU doctors in KSA hospitals	FM residents & primary healthcare (PHC) physicians in Qassim
Sample Size	n = 4,558	n = 189	n = 368	n = 359	n = 5,000	n = 155
Age	54.6% aged < 30 years	Mean 27.25 years (2.58)	42.4% aged 30–39 years	75.8% aged 25–34 years	Mean 45.23 years (12.12)	Mean 29.85 years (5.32)
Gender	Male 56.1%, Female 43.9%	Male 51.3%, Female 48.7%	Male 55.2%, Female 44.8%	Male 69.6%, Female 30.4%	Male 65.2%, Female 34.8%	Male 51.0%, Female 49.0%
Specialty / Professional Level	Medical interns 27.3%, residents 30.5%, consultants 16.6%, specialists 15.9%, service job 9.7%; all specialties included	FM residents only: PGY-1 27%, PGY-2 35.4%, PGY-3 37.6%	Medical interns 13.3%, residents 38.9%, GPs 26.1%, specialists 13.0%, consultants 8.7%	FM 16.7%, internal medicine 16.4%, EM 14.2%, paediatrics 9.7%; < 5 years practice 66.3%	EM 30.6%, FM 39.6%, ICU 29.8%; residents 10.7%, board certified 42.5%, consultants 26.4%	FM consultants 5.2%, FM specialists 35.5%, FM residents 44.5%, PHC physicians 14.8%

Result Synthesis

Unless otherwise stated, responses to five-point Likert items are reported as mutually exclusive categories and percentages are calculated on each study's total sample; positive, neutral, and negative categories therefore sum to 100% within each item.

An overview of the headline findings across all six studies and domains is presented in Table 3, and is expanded in the domain-specific subsections below.

**Table 3:** Synthesis matrix of headline findings across the six included studies. Values are % (n) of each study's total sample unless stated otherwise. NR, not reported. Items were measured with non-identical instruments and are not directly comparable across studies.

Study (N)	Adequate knowledge	Would assist / identify as a doctor	Confident to manage an IME	Prior IME-specific training	Medicolegal concern	Want more IME training
AlShamlan et al., 2021 (4,558) [18]	NR	Only 646 (15.0%) would not identify themselves	41.9% (1,907)	20.1% (915)	48.7% (2,217)	89.2% (4,068)
Alarifi & ALRowais, 2023 (189) [13]	NR	57.7% (109)	18.0% (34)	5.8% (11)	57.7% (109)	93.2% (176)
Hussien et al., 2024 (368) [19]	NR	64.2% (236) believe physicians should volunteer; 62.0% (228) had assisted	61.4% (226) felt adequately prepared	NR	61.1% (227)	67.6% (249)
Almadhyan et al., 2025 (359) [17]	50.7% (182)	66.8% (240)	16.8% felt fully capable	28.7% (103)	51.8% (186)	70.5% (253)
Hawati et al., 2025 (5,000) [16]	74.9% (3,745)	58.1% (2,903)	43.1% (2,156)	48.4% (2,421)	39.6% (1,979)	70.0% (3,497)
Almutairi & Sulaiman, 2025 (155) [12]	49.0% (76)	76.7% (119)	54.9% (85)	5.2% (8)	47.7% (74) would assist despite fear	88.4% (137)

Knowledge

Overall results in the included studies about how physicians in Saudi Arabia manage in-flight medical emergencies show varying degrees of knowledge of the health care providers. As per Almutairi and Sulaiman [12] (N = 155), almost half, 49.0% (n = 76), of the participants had adequate knowledge about IMEs, while the remaining 51.0% (n = 79) had inadequate knowledge. According to Hawati et al. [16] (N = 5,000), there is an overall 74.9% (n = 3,745) of participants possessing adequate knowledge, but with good variability by region and specialty. The performance gap was particularly noted in advanced airway management and cardiac arrest recognition, as well as aviation physiology. Likewise, Almadhyan et al. [17] (N = 359) reported that while 76.6% (n = 275) recognized the impact of cabin pressure on oxyhemoglobin saturation, only 54.6% (n = 196) correctly identified that patients with an acute asthma exacerbation benefit from altitude restriction. Most physicians aid during IMEs, which may demonstrate their level of practical knowledge and urges. In this regard, emergency physicians reported the highest level of willingness and confidence [18].

Awareness

It has been observed that the healthcare providers lack sufficient knowledge about aviation physiology and onboard equipment. A recent study by Alarifi and AlRowais [13] (N = 189) showed that based on the survey results, 73.0% (n = 138) of the participants stated that they were not aware of the available medical equipment on board. Additionally, 65.9% (n = 124) were unaware of the training level of the crew, and 57.7% (n = 109) stated that they were unaware of the interagency protocols. According to the findings of Almadhyan et al. [17] (N = 359), 76.6% (n = 275) of respondents recognized the impact of cabin pressure on SpO_2_, but only 54.6% (n = 196) accurately identified the altitude-related risk for asthmatic patients, with 45.4% (n = 163) answering incorrectly. Moreover, only 48.7% (n = 175) reported adequate awareness of available onboard medical supplies and 39.8% (n = 143) of onboard medications, and only 32.9% (n = 118) were aware of the collaboration protocols between cabin crew, ground-based control and onboard volunteers. According to Hawati et al. [16] (N = 5,000), 75.2% (n = 3,759) were aware that cabin pressure decreases systemic oxyhemoglobin saturation and 82.5% (n = 4,124) identified low cabin humidity, but only 50.2% (n = 2,512) identified confusion as the most common symptom of decompression sickness. Regarding medical kit contents, Almadhyan et al. [17] reported that 84.4% (n = 303) recognized epinephrine and 66.9% (n = 240) injectable dextrose, while fewer identified aspirin, 61.3% (n = 220), or anticonvulsants, 51.3% (n = 184). According to Almutairi and Sulaiman [12] (N = 155), 81.3% (n = 126) knew that cabin pressure decreases oxyhemoglobin saturation and 64.5% (n = 100) that cabin humidity is lower than at ground level; 87.1% (n = 135) identified the sphygmomanometer and 96.8% (n = 150) epinephrine among medical kit contents, but only 37.4% (n = 58) were aware that a laryngoscope is available.

Attitude

The attitudes of healthcare professionals toward volunteering in in-flight emergencies reveal a mix of willingness and concerns. In a study by AlShamlan et al. [18] (N = 4,558), 48.7% (n = 2,217) agreed or strongly agreed that they are fearful of the legal implications of emergency medicine, but most were willing to identify as doctors. Emergency department doctors had the most positive attitudes. The study by Alarifi and AlRowais [13] (N = 189) showed that 57.7% (n = 109) are frightened by the legalities and 46.0% (n = 87) have the belief that they will not do it if a similar provider has already done it. After all, the study showed that 93.2% (n = 176) agreed on the need for more IME training. According to Hussien et al. [19] (N = 368), 64.2% (n = 236) believed that physicians should volunteer. In addition, participation was motivated by 67.2% (n = 247) of responders, although 62.5% (n = 230) acknowledged facing barriers to volunteering. On the other hand, 60.1% (n = 221) believed that airline staff are well-trained. As per Almadhyan et al. [17] (N = 359), 51.8% (n = 186) cited legal concerns as a barrier, 17.0% (n = 61) strongly agreed they would refrain if another specialist had already stepped forward, and 70.5% (n = 253) said they require more IME training. According to a study conducted by Hawati et al. [16] (N = 5,000), 58.1% (n = 2,903) agreed or strongly agreed that they would identify themselves as a doctor during an IME. Approximately 39.6% (n = 1,979) of the participants were concerned about any medicolegal implications. Most of the participants, about 70.0% (n = 3,497), acknowledged the need for more training on IMEs. Finally, in Almutairi and Sulaiman [12] (N = 155), 49.0% (n = 76) of respondents agreed that they would probably volunteer and 51.0% (n = 79) were probably willing to help on the scene even if someone else was helping. Furthermore, 88.4% (n = 137) agreed that there is a need for more training, and 47.7% (n = 74) indicated they would probably or definitely help even if they feared legal consequences, while 35.5% (n = 55) would not.

Confidence

Data on the confidence of volunteers in dealing with in-flight emergencies shows considerable variation and generally low confidence. According to AlShamlan et al. [18] (N = 4,558), only 41.9% (n = 1,907) experienced adequate training regarding IME, whereas 56.3% (n = 2,568) reported being adequately trained at ground level; emergency physicians display the highest level of confidence, and psychiatrists and radiologists show the lowest levels. Alarifi and ALRowais [13] (N = 189) indicated that 18.0% (n = 34) agreed they would feel confident responding to an IME and providing competent care, 46.0% (n = 87) were undecided, and 36.0% (n = 68) disagreed. Hussien et al. [19] (N = 368) reported that 61.4% (n = 226) felt adequately prepared to respond, while those aged under 30 years scored the highest confidence (54.85 ± 7.03). Furthermore, previous IME experience was significantly related to confidence levels (P < 0.001). Almadhyan et al. [17] show that 48.7% felt their medical education was fair but only 16.8% felt fully competent to handle IMEs. In their study, Hawati et al. [16] (N = 5,000) presented data indicating that 43.1% (n = 2,156) of respondents agreed or strongly agreed that they felt confident responding to an IME, with only 7.7% (n = 388) strongly agreeing. Lastly, in Almutairi and Sulaiman [12] (N = 155), 16.8% (n = 26) felt definitely and 38.1% (n = 59) probably confident to volunteer in an IME, while 17.4% (n = 27) felt probably not confident, 14.2% (n = 22) definitely not, and 13.5% (n = 21) were unsure. Regarding whether their medical education had sufficiently equipped them to handle an IME, 16.8% (n = 26) answered definitely and 30.3% (n = 47) probably, whereas 27.7% (n = 43) answered probably not, 14.2% (n = 22) definitely not, and 11.0% (n = 17) were unsure. Moreover, over half, 53.0% (n = 82), felt they knew little or nothing about what onboard equipment is available.

Willingness to Assist

Research on willingness to help in in-flight medical emergencies suggests that medical volunteers are generally willing to help, although several factors may influence their willingness. According to AlShamlan et al. [18] (N = 4,558), the large majority would identify themselves as doctors in an IME situation, with only 646 participants (15.0%) reporting that they would not. Significant predictors for willingness to assist included being male (odds ratio, OR, 1.6), having previous IME (OR 1.5), having ever done life support (OR 2.4), frequent traveling, practicing in the Central region and having the European or Arab board. After studying the factors influencing the ability to aid, Alarifi and AlRowais [13] (N = 189) found that 57.7% (n = 109) would identify and help. Being able to travel two or more times a year (OR 2.108, P = 0.015) and perceived confidence (OR 3.530, P = 0.001) were significant factors, whereas being male gender, older age and high training did not impact these findings. According to Hussien et al. [19] (N = 368), 67.2% (n = 247) volunteered out of motivation. In addition, 67.1% (n = 247) had encountered an IME, and 62.0% (n = 228) of the whole sample had assisted in one. Having previously encountered an IME was significantly associated with higher scores (P<0.001). Almadhyan et al. [17] (N = 359) found that 66.8% (n = 240) would offer help although 17.0% (n = 61) would not if another specialist helped first. A study by Hawati et al. [16] (N = 5,000) found that 58.1% (n = 2,903) agreed or strongly agreed that they would identify themselves as a doctor during an IME, while 26.8% (n = 1,341) would stay out if someone else was already helping. Finally, participants in Almutairi and Sulaiman [12] (N = 155) reported a willingness to volunteer of 76.7% (n = 119; 27.7%, n = 43, definitely and 49.0%, n = 76, probably), and 47.7% (n = 74) would still offer help despite fear of legal consequences.

Preparedness

According to AlShamlan et al. [18] (N = 4,558), while only 20.1% (n = 915) of participants ever attended an IME course, 34.4% (n = 315) of those who attended were in the high-confidence group compared with 11.0% (n = 402) of non- attendees. The difference was statistically significant (P < 0.01), suggesting this course may enhance participant confidence. On the other hand, Alarifi and ALRowais [13] (N = 189) reported that 94.2% (n = 178) of people have never received IME-specific training. In addition, 40.7% (n = 77) of participants believed their medical education did not adequately prepare them for IME scenarios. There is a clear lack of aviation-specific preparation. According to Hussien et al. [19] (N = 368), only 34.2% (n = 126) had BLS skills, only 13.6% (n = 50) had ACLS certification, and 58.6% (n = 215) said lack of specific training was a factor. According to Almadhyan et al. [17] (N = 359), it was observed that 65.7% (n = 236) of the participants had never contributed to an IME; however, 28.7% (n = 103) had IME training. Additionally, 96.1% (n = 345) had general life support training, while 70.5% (n = 253) of them expressed the need for training. According to Hawati et al. [16] (N = 5,000), 48.4% (n = 2,421) had received training on in-flight emergencies, while 70.0% (n = 3,497) acknowledged the need for further training. The study showed significant gaps in onboard equipment knowledge and crew collaboration protocols. Almutairi and Sulaiman [12] (N = 155) indicated that 94.8% (n = 147) of Qassim healthcare staff had never attended any type of IME-specific course, although 98.7% (n = 153) had done BLS and 74.8% (n = 116) had done ACLS. Additionally, only 5.2% (n = 8) had received IME-specific training, while 65.8% (n = 102) expressed a very strong need for this training.

Barriers

AlShamlan et al. [18] (N = 4,558) reported that almost 48.7% (n = 2,217) of the volunteers were discouraged because of the medicolegal issues, and it was further complicated with lack of skills as well as incompetence of diagnosis. Moreover, Alarifi and ALRowais [13] (N = 189) found a rate of 57.7% (n = 109) of medicolegal concerns, inadequate familiarity, and inadequate training. A study conducted by Hussien et al. [19] (N = 368) revealed that 58.6% (n = 215) of volunteers indicated they were not given specialized training. Furthermore, 61.1% (n = 227) cited legal issues, usually stemming from pressure-cooker circumstances and restricted resources. Almadhyan et al. [17] (N = 359) reported about 51.8% (n = 186) of possible medicolegal concerns and uncertainties about collaboration protocols and the onboard resource. According to Hawati et al. [16] (N = 5,000), 39.6% (n = 1,979) of individuals were concerned with the medicolegal implications amid the lack of training specifically on IME as well as environmental constraints. In addition, Almutairi and Sulaiman [12] (N = 155) reported low self-rated competence, with only 16.8% (n = 26) feeling definitely confident to volunteer and 53.0% (n = 82) knowing little or nothing about available onboard equipment, alongside widespread medicolegal apprehension. Furthermore, there was a limited understanding of resources onboard and teamwork.

Factors Affecting Competence and Readiness

Several studies have identified main predictors for physicians' willingness, confidence and knowledge for management of in-flight medical emergencies in Saudi Arabia. As revealed by the findings of AlShamlan et al. [18], male physicians reported higher willingness (12.7%, n = 326, vs. 7.9%, n = 158) and confidence (18.3%, n = 468, vs. 12.4%, n = 249) than female physicians, and previous IME experience was associated with higher willingness (14.5%, n = 220, vs. 8.7%, n = 264) and confidence (22.8%, n = 346, vs. 12.2%, n = 371). Life support course attendance, frequent traveling and practicing in the Central region were also associated with significantly higher levels of willingness and confidence (P < 0.05 and P < 0.01). According to their report, only 12.1% (n = 303) of those with less than five years of experience had high confidence versus 47.1% of those with 15 or more years. According to Almutairi and Sulaiman [12], assessment of knowledge levels did not reveal significant differences between the demographic characteristics of the study participants. However, it was discovered that those with income levels less than 10,000 SAR tended to have inadequate knowledge levels. Correct responses from subjects ranged from 19.4% to 80.6%. In Almadhyan et al. [17], adequate knowledge was significantly associated with area of practice (P = 0.020), duration of emergency department experience (P = 0.041), prior experience assisting during an IME (P = 0.021), and prior IME training (P < 0.001); notably, participants without prior IME experience or training were more likely to demonstrate adequate knowledge, which the authors attributed to self-selection bias. According to Hussien et al. [19], physicians who had previously encountered an IME scored higher on the attitude scale than those who had not (54.64 ± 7.81 vs. 49.30 ± 7.10, P < 0.001). In addition, younger doctors, younger than 30 years, had the highest confidence (54.85 ± 7.03), while doctors with less than five years of experience scored better than doctors with higher experience (54.69 ± 7.16 vs. 52.25 ± 5.75, P = 0.039). Most of the participants noted barriers like lack of training (58.6%, n = 215) and legal issues (61.1%, n = 227), with 67.6% (n = 249) needing more training. According to a study conducted by Hawati et al. [16], it was found that 76.8% (n = 2,504) of males, as compared to 71.3% (n = 1,242) of females, were able to show adequate knowledge (P = 0.001). In addition, physicians practicing in the Middle region showed the highest rate of adequate knowledge (90.8%, n = 1,325; P = 0.005). By specialty, ICU specialists (80.5%, n = 1,200) and family medicine physicians (76.9%, n = 1,521) obtained the highest scores (P = 0.0001). In a study by Alarifi and ALRowais [13], frequent travelers, defined as two or more times per year, were more likely to offer care (OR 2.108, P = 0.015), as were those who had more perceived confidence (OR 3.530, P = 0.001). Male gender, older age, higher level of training, and prior IME training had no significant association with offering care.

Risk of Bias

Most of the studies included had a moderate risk of bias, while much lower risks were observed in others. AlShamlan et al. [18], Alarifi and ALRowais [13], Hawati et al. [16], as well as Almutairi and Sulaiman [12] were rated as having a moderate risk of bias due to unclear reporting for some methodological aspects such as measurement of confounding factors and outcome assessment. On the contrary, there is a low risk of bias in Hussien et al. [19] and Almadhyan et al. [17] due to their clear definition of inclusion criteria, appropriateness of measurement strategies, and strategies for analysis. The full appraisal is presented in Table 4. The moderate ratings were driven almost entirely by unclear identification of confounding factors and unclear validity of exposure and outcome measurement, rather than by deficiencies in sampling frames, statistical analysis, or reporting of setting, which were rated adequate in every study. Because the domains most often rated unclear concern measurement and confounding, the narrative findings reported below should be read as indicative rather than precise, and part of the between-study variation in knowledge and confidence is likely to reflect differences in instrument construction and scoring thresholds rather than true differences in physician competence.

**Table 4:** Risk of Bias Assessment using the Joanna Briggs Institute (JBI) critical appraisal tool.

JBI Item	AlShamlan et al. (2021) [18]	Alarifi & ALRowais (2023) [13]	Hussien et al. (2024) [19]	Almadhyan et al. (2025) [17]	Hawati et al. (2025) [16]	Almutairi & Sulaiman (2025) [12]
1. Were the criteria for inclusion in the sample clearly defined?	Yes (Low)	Yes (Low)	Yes (Low)	Yes (Low)	Yes (Low)	Yes (Low)
2. Were objective, standard criteria used for measurement of the condition?	Unclear	Unclear	Unclear	Yes (Low)	Yes (Low)	Unclear
3. Was the exposure measured in a valid and reliable way?	Unclear	Unclear	Yes (Low)	Yes (Low)	Unclear	Yes (Low)
4. Were the outcomes measured in a valid and reliable way?	Unclear	Unclear	Yes (Low)	Yes (Low)	Unclear	Unclear
5. Were confounding factors identified?	Unclear	Unclear	Yes (Low)	Unclear	Unclear	Unclear
6. Were strategies to deal with confounding factors stated?	Yes (Low)	Yes (Low)	Yes (Low)	Unclear	Yes (Low)	Unclear
7. Was appropriate statistical analysis used?	Yes (Low)	Yes (Low)	Yes (Low)	Yes (Low)	Yes (Low)	Yes (Low)
8. Were the study subjects and the setting described in detail?	Yes (Low)	Yes (Low)	Yes (Low)	Yes (Low)	Yes (Low)	Yes (Low)
OVERALL RISK OF BIAS	Moderate	Moderate	Low	Low	Moderate	Moderate

Discussion

To our knowledge, this systematic review is the first to present a comprehensive picture of physicians' preparedness for in-flight medical emergencies in Saudi Arabia. The main implication is not only that knowledge is incomplete, but that there is also a consequential disconnect between willingness and readiness to operate. Doctors are generally receptive to helping, but many lack an understanding of aviation physiology, the material and limitations of medical resources onboard, the roles of the volunteer, cabin crew, ground medical support and captain and the medico-legal aspects of providing care. Willingness is attitudinal; readiness is knowledge, context-specific, calibrated confidence, adaptability, communication and decision-making under extreme resource constraints. In international surveys, physicians have consistently expressed positive attitudes towards helping behavior, but lack of knowledge of airline protocols, equipment and the actual implementation of in-flight care [22,23]. So, the Saudi results can be interpreted more as a problem of systems-preparedness rather than lack of overall clinical competence.

Systems Preparedness Versus Clinical Competence

The gap observed is clinically plausible as an aircraft cabin creates non-routine tasks from otherwise familiar emergencies. Standard diagnostic routines have limitations due to noise, limited access to the patient, poor lighting, limited monitoring, lack of definitive imaging and laboratory testing, and delayed access to hospital care. Thus, effective performance relies on quick and accurate syndromic evaluation, prioritization, judicious deployment of limited equipment, and conveying uncertainty. In retrospect, airline data reveals that symptom complexes of transient loss of consciousness, gastrointestinal symptoms, respiratory complaints, and cardiovascular or neurologic presentation dominate events, and a minority of events result in high-stakes diversion decisions [24,25]. Multinational evidence confirms that serious neurologic and cardiac events are especially to have an impact and that physician involvement is focused on complex cases [26]. Therefore, the curriculum for preparedness should focus on common presentations and thresholds for decision-making, and not encyclopedic lists of uncommon diagnoses.

Life-Support Certification Is Not a Proxy for In-Flight Competence

The review also indicates that generic life-support certification should not be treated as a proxy for in-flight competence. Basic and advanced resuscitation courses are still crucial but are typically held in a setting that has known teams, set-up carts, space and straight escalation routes. Of these assumptions, none can be taken over without critical consideration of commercial flight. Aviation-specific capability involves appreciation of decreased ambient pressure and humidity, finding and safely using oxygen, automated external defibrillators (AEDs), and emergency drugs, adjusting examination and procedural techniques, knowing when continued observation is reasonable, and communicating a concise recommendation, not taking on operational authority, to divert an aircraft. These skills are worthy of having a specific domain in the curriculum. The evidence from Saudi medical students indicates that the gap in preparedness started not after postgraduate practice, which means that it is needed to take a longitudinal approach to education rather than a remedial approach [27].

Specialty, Prior Exposure, and Demographic Predictors

Relationships between previous exposure, specialty, training and confidence need to be understood in the context of contextual familiarity. Family physicians may have a breadth of knowledge, risk-stratification and continuity-oriented judgment, while emergency medicine and critical care physicians regularly work with undifferentiated illness, time pressure, incomplete information and rapid disposition decisions. But not every specialty is ready for the aviation environment. On the other hand, doctors working in non-acute specialties can work well if they have well-defined roles, an easily accessible algorithm, and back-up support. There are inconsistent demographic associations, which should make one wary of sex, age or seniority targeting of education. It is important to note that apparent differences may be due to exposure inequities, frequency of travel, specialty mix or selection for training and not necessarily reflect readiness for training. Training should thus be competency-based and widely accessible, and in addition, should be deeper for frequent travelers and clinicians who may be required during an international flight.

Medicolegal Uncertainty

A specific explanatory factor that arose was the medicolegal concern. Fear of liability can cause a person to be less likely to volunteer, be less likely to admit to being a physician, or give a suggestion too guarded. However, there is often legal uncertainty coupled with a lack of clarity around who is leading care and what is required in terms of documentation. Studies of physicians' Good Samaritan behavior outside the scope of their usual practice have revealed that perceived knowledge of legal protection is related to increased likelihood of intervening [28]. Education should not offer vague reassurance. It should include an explanation in simple terms of the applicable Saudi framework, the potential relevance of the registration and location of the aircraft, the extent of protection where impairment or grossly negligent conduct is found, the importance of gaining consent wherever possible and the importance of recording findings and advice. International legal rules vary, so the best educational message is to be honest, show credentials, work within scope of competence, work with crew and ground support, suggest not dictating operational decisions and document the encounter.

Simulation-Based and Competency-Based Training

The most important actionable implications are the need for a nationally standardized educational pathway based on simulations. Brief simulation has been shown to enhance aviation-emergency knowledge and confidence in learners [29], and randomized evidence suggests that a point-of-care electronic aid modestly improves the performance of non-emergency residents in simulated in-flight scenarios [30]. Family medicine education programs have also shown that it is possible to integrate aviation emergencies into the residency curriculum [31]. Higher fidelity programs using realistic cabins have demonstrated improvements in knowledge and self-rated ability of emergency medicine trainees [32], and peer-reviewed simulation scenarios offer flexible models for team leadership, cardiac arrest, and tension pneumothorax [33]. These are typically small studies with short-term outcomes, but they are in favor of experiential learning over passive delivery.

The national curriculum should be concise and sufficiently detailed to measure performance. A core module might involve pre-course digital learning, a brief workshop, and recurrent micro-simulation, structured around the following components.

Aviation physiology: Cabin pressurization and altitude, hypoxia, reduced humidity, and gas expansion in body cavities.

Common in-flight syndromes: Syncope, hypoglycemia, anaphylaxis, asthma, seizure, chest pain, stroke, and cardiac arrest.

Resources: Systematic use of the emergency medical kit, operation of onboard oxygen and the automated external defibrillator, medication choice constrained by kit contents, and infection control.

Communication and escalation: Structured handover to cabin crew, consultation with ground-based medical support, and the formulation of a diversion recommendation without assuming operational authority.

Scenario realism: Noise, restricted space, language barriers, incomplete history, and variable equipment across carriers.

Assessment: Observable critical actions, diagnostic prioritization, communication quality, correct equipment use, and calibration of confidence against measured performance rather than self-rated confidence alone.

Recurrence: Scheduled refresher training, since in-flight emergencies are rare events and skill decay is predictable.

Physician-Crew Integration

Solving the problem by educating doctors alone would only solve half the equation, however. An in-flight response is a distributed safety process whereby the cabin crew makes assessments, controls access to equipment and communications, coordinate with the flight deck and implements airline procedures. Based on the results of the cabin-crew study, positive attitudes to first aid and commitment to work are linked to higher self-efficacy in providing first aid [34]. Joint physician and crew simulation could thus create a shared mental model, where the crew maintains operational control, the medical volunteer makes clinical assessments and recommendations, aviation-aware ground medical support provides consultation, and the captain makes the diversion decision. This type of structure minimizes role conflict and can make the task bounded and supported, which can lead to increased willingness. Airlines need to issue a short orientation card to the point of response that includes what equipment is available and how to communicate, what documentation is required, and who will be doing what.

*Alignment With* *Existing Aviation Governance*

Such initiatives could be incorporated into the existing aviation governance structure, instead of having a parallel medical program in Saudi Arabia. Operator procedures, required equipment, cabin-crew training and recurrent preparedness are already addressed in the current General Authority of Civil Aviation regulations. Through the cooperation of the aviation regulator, health authorities, training organizations, airlines and ground medical support providers, this infrastructure could be transformed into a nationally consistent competency framework. The Saudi Commission for Health Specialties may be able to approve an accredited short course for continuing professional development, and medical schools could incorporate a basic module in emergency medicine or public health courses. Physicians might be invited to participate in a select number of crew simulations, and airlines might be able to harmonize quick-reference materials. Standardization should be preferred to airline-specific memorization, but it is also important to train that the kit content and procedure can differ between airlines and jurisdictions.

Limitations

There is a limited degree of certainty in the evidence. The synthesis reflects reported knowledge, attitudes, and confidence, as all included studies were cross-sectional, and the majority used a questionnaire. They may have been more likely to respond to an aviation emergency, and online and convenience recruitment may have led to overreporting of willingness or previous assistance, reflecting recall and social-desirability bias. Meta-analysis was not possible due to differences in questionnaires and thresholds, populations and regional coverage, which also make direct comparisons difficult. Some studies share a common conceptual or questionnaire origin, and several were derived from the same parent instruments, which limits their methodological independence. More importantly, the six studies cannot be assumed to represent six independent respondent pools. Almutairi and Sulaiman and Alarifi and ALRowais both surveyed Saudi family medicine trainees, and several studies recruited through the same national registry of the Saudi Commission for Health Specialties or through overlapping electronic professional networks. Similar populations may therefore have contributed to more than one study, so consistent findings across studies may partly reflect repeated sampling of comparable respondents rather than independent replication, and the narrative synthesis should not be read as if it aggregated six distinct populations. In addition, although screening and data extraction were performed independently in duplicate, inter-rater agreement was not quantified, so the reproducibility of the selection and appraisal decisions cannot be expressed statistically. None of the studies included in this research measured physician readiness in relation to passenger outcomes, quality of care or appropriateness of diversion. Thus, training or pre-exposure and readiness relationships cannot prove causality. The limited number of eligible studies, the English language restriction and the lack of perspectives from the airline, the cabin crew and the ground support further limit the comprehensiveness of the conclusions.

Implications for Practice

The Saudi evidence, overall, presents a picture of a motivated workforce of physicians who are constrained in their readiness by context-specific gaps in knowledge, unclear boundaries of practice, lack of training in aviation medicine, and ambiguity about medicolegal issues. The right answer is not for every traveling physician to be an aviation-medicine specialist, but to give every one of them a minimum, nationally standard capability for safe assessment, use of resources, teamwork, and escalation. Willingness would improve reliable performance with competency-based education, realistic interprofessional simulation, point-of-care decision support, clearer legal and operational guidance, and a national outcomes registry. These measures are all directly related to the growing Saudi aviation industry and constitute a real-life patient-safety agenda for the regulators, the educational institutions, the airlines and even the healthcare organizations.

Implications for Future Research

Future research needs to build on descriptive surveys and move toward effectiveness and implementation studies. Cluster randomized trials or stepped wedge trials might be used to compare didactic instruction, simulation, digital decision support, and blended learning, as well as to assess outcomes immediately and at six to 12 months. There is a need for studies to evaluate critical-action completion, teamwork, diversion recommendations, and transfer to practice, in addition to knowledge and confidence. A confidential national IME registry created in cooperation with the airlines and ground medical support services would be particularly helpful. Structured records can describe case mix, volunteer involvement, interventions and diversion decisions, as demonstrated by airline studies [24-26,35]. A Saudi registry could correlate volunteer qualifications and onboard actions to operational and clinical outcomes and safeguard passenger privacy. This data would enable the training priorities and kit policies to be based on the local event patterns and not assumptions.

## Conclusions

Saudi physicians are highly willing to help in an in-flight medical emergency; however, they have limited knowledge of the aviation environment, inconsistent aviation-specific knowledge, lack of confidence, and concerns regarding medicolegal issues. There seems to be a benefit to prior exposure and focused training for preparedness.

To make willingness a reality and turn it into safe and effective in-flight care, standardized national education, simulation-based training, clear legal guidance, and increased cooperation between healthcare institutions, airlines and aviation authorities are therefore likely required.

## References

[1] (2026). Air transport, passengers carried. https://data360.worldbank.org/en/indicator/WB_WDI_IS_AIR_PSGR.

[2] Potin M, Carron PN, Genton B (2024). Injuries and medical emergencies among international travellers. J Travel Med.

[3] Hu JS, Smith JK (2021). In-flight medical emergencies. Am Fam Physician.

[4] Peterson DC, Martin-Gill C, Guyette FX (2013). Outcomes of medical emergencies on commercial airline flights. N Engl J Med.

[5] Muhm JM, Rock PB, McMullin DL (2007). Effect of aircraft-cabin altitude on passenger discomfort. N Engl J Med.

[6] Martin-Gill C, Doyle TJ, Yealy DM (2018). In-flight medical emergencies: A review. JAMA.

[7] Battineni G, Arcese A, Chintalapudi N, Di Canio M, Sibilio F, Amenta F (2024). Approaches to medical emergencies on commercial flights. Medicina (Kaunas).

[8] Dowdall N (2000). "Is there a doctor on the aircraft?" Top 10 in-flight medical emergencies. BMJ.

[9] Kim JH, Choi-Kwon S, Park YH (2017). Comparison of inflight first aid performed by cabin crew members and medical volunteers. J Travel Med.

[10] Shi R, Jiang W, Yang J (2024). Characteristics of in-flight medical emergencies on a commercial airline in mainland China: Retrospective study. JMIR Public Health Surveill.

[11] Gendreau MA, DeJohn C (2002). Responding to medical events during commercial airline flights. N Engl J Med.

[12] Almutairi R, Sulaiman AA (2025). Assessment of family medicine physicians' and residents' knowledge in managing in-flight medical emergencies: A cross-sectional study from Qassim, Saudi Arabia. Cureus.

[13] Alarifi AS, ALowais N (2023). Assessing family medicine residents' knowledge, attitudes, and confidence in managing in-flight medical emergencies in Riyadh, Saudi Arabia. Cureus.

[14] Page MJ, McKenzie JE, Bossuyt PM (2021). The PRISMA 2020 statement: An updated guideline for reporting systematic reviews. BMJ.

[15] Moola S, Munn Z, Tufanaru C (2020). Systematic review of etiology and risk. JBI Manual for Evidence Synthesis.

[16] Hawati SM, Binobaid F, Alsaeigh A (2025). Assessing emergency medicine, family medicine, and ICU doctors' knowledge, confidence, and attitude in managing in-flight medical emergencies in the Kingdom of Saudi Arabia hospitals: A cross-sectional study. Cureus.

[17] Almadhyan A, Almuteri D, Alsenidi S, Alazmi M, Rajab R, Alghamdi S, Alkuraydis S (2025). Emergency at altitude: Exploring physicians' knowledge, preparedness, and perspectives on in-flight medical emergencies in Saudi Arabia. World J Emerg Med.

[18] AlShamlan NA, AlOmar RS, Alrayes MM (2021). "Is there a doctor on board?": Willingness and confidence of physicians in the Kingdom of Saudi Arabia in assisting with in-flight medical emergencies. BMC Emerg Med.

[19] Hussien AK, Aljaghwani SA, Albarrak IM (2024). Exploring the nature and challenges among physicians in Saudi Arabia responding to an inflight medical emergency: A cross-sectional survey. Cureus.

[20] Katzer RJ, Duong D, Weber M, Memmer A, Buchanan I (2014). Management of in-flight medical emergencies: are senior medical students prepared to respond to this community need?. West J Emerg Med.

[21] Ng WL, Abdullah N (2020). Knowledge, confidence and attitude of primary care doctors in managing in-flight medical emergencies: A cross-sectional survey. Singapore Med J.

[22] Hinkelbein J, Neuhaus C, Böhm L, Kalina S, Braunecker S (2017). In-flight medical emergencies during airline operations: A survey of physicians on the incidence, nature, and available medical equipment. Open Access Emerg Med.

[23] Chatfield E, Bond WF, McCay B (2017). Cross-sectional survey of physicians on providing volunteer care for in-flight medical events. Aerosp Med Hum Perform.

[24] Sand M, Bechara FG, Sand D, Mann B (2009). Surgical and medical emergencies on board European aircraft: A retrospective study of 10189 cases. Crit Care.

[25] Mahony PH, Myers JA, Larsen PD, Powell DM, Griffiths RF (2011). Symptom-based categorization of in-flight passenger medical incidents. Aviat Space Environ Med.

[26] Alves PM, Kumar KR, Devlin J, Nerwich N, Rotta AT (2025). In-flight medical events on commercial airline flights. JAMA Netw Open.

[27] Alsulimani L, Masri T, Abdulhalim A (2024). The knowledge, confidence and attitudes of medical students in managing in-flight medical emergencies. Signa Vitae.

[28] Garneau WM, Harris DM, Viera AJ (2016). Cross-sectional survey of Good Samaritan behaviour by physicians in North Carolina. BMJ Open.

[29] Padaki A, Redha W, Clark T (2018). Simulation training for in-flight medical emergencies improves provider knowledge and confidence. Aerosp Med Hum Perform.

[30] Nadir NA, Cook CJ, Bertino RE (2019). Impact of an electronic app on resident responses to simulated in-flight medical emergencies: Randomized controlled trial. JMIR Med Educ.

[31] Udezi V, Day P, Gimpel N (2021). Training family medicine residents to prevent and respond to in-flight emergencies. Med Sci Educ.

[32] Kommor MB, Miller KN, Powell TL (2023). A first-class simulation: In-situ in-flight medical emergencies curriculum for emergency medicine residents aboard a commercial airliner. Cureus.

[33] Hailey C, Lei C, Lawrence L (2020). In-flight emergency: A simulation case for emergency medicine residents. MedEdPORTAL.

[34] Yu YC, Liang JC (2021). Relationships among affect, hardiness and self-efficacy in first aid provision by airline cabin crew. Int J Environ Res Public Health.

[35] Hung KK, Cocks RA, Poon WK, Chan EY, Rainer TH, Graham CA (2013). Medical volunteers in commercial flight medical diversions. Aviat Space Environ Med.

